# Potential mechanism of 25R-inokosterone in the treatment of osteoporosis: Based on bioinformatics and molecular dynamics model

**DOI:** 10.1097/MD.0000000000043585

**Published:** 2025-08-01

**Authors:** Jun Tan, Liwei Huo, Mengting Hu, Yidong Xu, Weinian Liu, Guangwei Wang, Enlong Fu, Jiling Liu

**Affiliations:** a Guangdong Provincial People’s Hospital’s Nanhai Hospital, Foshan, Guangdong Province, China; b Guangzhou Orthopedic Hospital, Guangzhou University of Chinese Medicine, Guangzhou, Guangdong Province, China; c The Shenzhen Luohu Hospital Group-Shenzhen Luohu Traditional Chinese Medicine Hospital, Shenzhen, Guangdong Province, China; d The Third Clinical College, Guangzhou University of Chinese Medicine, Guangzhou, Guangdong Province, China; e The First Clinical College, Guangzhou University of Chinese Medicine, Guangzhou, Guangdong Province, China.

**Keywords:** 25R-inokosterone, molecular docking, molecular dynamics simulations, network pharmacology, osteoporosis

## Abstract

25R-inokosterone, a sterone derived from *Achyranthes bidentata* Bl., has potential anti-osteoporotic effects. However, the underlying mechanism of 25R-inokosterone in the treatment of osteoporosis is unknown. The aim of this study was to investigate the molecular mechanism of 25R-inokosterone’s anti-osteoporosis by network pharmacology and molecular docking. First, the structural formula of 25R-inokosterone was obtained by PubChem and potential targets were predicted by SwissTargetPredictive. The genes of osteoporosis (OP) were obtained by GeneCards, OMIM, therapeutic target database and database of gene–disease associations. Protein interactions and functional enrichment of potential targets were analyzed using STRING, gene ontology, and Kyoto encyclopedia of genes and genomes pathway databases. Finally, the hub targets were identified by network pharmacology, and the interaction of their hub targets with 25R-inokosterone was verified by molecular docking and molecular dynamics simulations. The results showed that 43 potential targets were associated with the mechanism of 25R-inokosterone for OP treatment. Enrichment analysis showed that hub genes were mainly associated with prolactin signaling pathway, aldosterone-regulated sodium reabsorption, phosphatidylinositol-3 kinase-Akt signaling pathway, and mTOR signaling pathway. Molecular docking showed that 25R-inokosterone was associated with PIK3CA, MTOR, TNF, MAPK3, CDK2, and NTRK1 with good affinity. Among them, 25R-inokosterone has the highest affinity with PIK3CA/MTOR, which is supported by molecular dynamics simulations. Our findings suggested that 25R-inokosterone may against OP by regulating multiple targets such as PIK3CA, MTOR, TNF, MAPK3, CDK2, and NTRK1 and phosphatidylinositol-3 kinase/AKT/mTOR signaling pathway. These findings provide a theoretical basis for the treatment of OP.

## 1. Introduction

Osteoporosis (OP) is a common metabolic bone disease in middle-aged and older adults, characterized by a decrease in bone mass, leading to bone weakness increased susceptibility to fracture decreased bone density.^[1]^ It is estimated that approximately 200 million people worldwide suffer from osteoporosis, nearly 22 million women and 5.5 million men in Europe and 10 million in the United States, and the number continues to rise.^[2]^ In 2017 to 2018, in the United States, the prevalence of osteoporosis in the femoral neck and lumbar spine was 12.6% in adults aged 50 and older, with a prevalence of 9.6% in women, compared to a prevalence of only 4.4% in men. In particular, the prevalence of age-adjusted low bone mass in the femoral neck or lumbar spine was 43.1%, and the prevalence in adults aged 50 to 64 years was 39.3%, while the prevalence in adults aged 65 years and older rose to 47.5%.^[3]^ Antiresorptive drugs such as bisphosphonates and SERMs, as well as anabolic drugs that stimulate bone formation, are the current treatments for osteoporosis. Despite the effectiveness of these drugs, severe side effects and loss of efficacy still limit the long-term use of a single drug.^[4]^

*Achyranthes bidentata* Bl. is the dried root of *A bidentata* Bl., family Amaranthaceae, which has the effects of expelling blood stasis and clearing up menstruation, tonifying the liver and kidneys, strengthening the bones and tendons, inducing diuresis, clearing up drenching and inducing the blood to flow downward.^[5]^ *A bidentata* Bl. mainly contains 25R-inokosterone, 25S-inokosterone, β-ecdysterone, red amaranth sterones, and other sterones.^[6,7]^ Pharmacological studies have shown that sterones have analgesic and anti-inflammatory effects, anti-osteoporosis, and hypoglycemic effects.^[8–10]^ The underlying mechanisms of 25R-inokosterone’s ability to prevent osteoporosis, nevertheless, remain mainly unclear. Here, we concentrate on the possible molecular mechanisms of 25R-inokosterone as an effective osteoporosis treatment.

**Figure 1.**
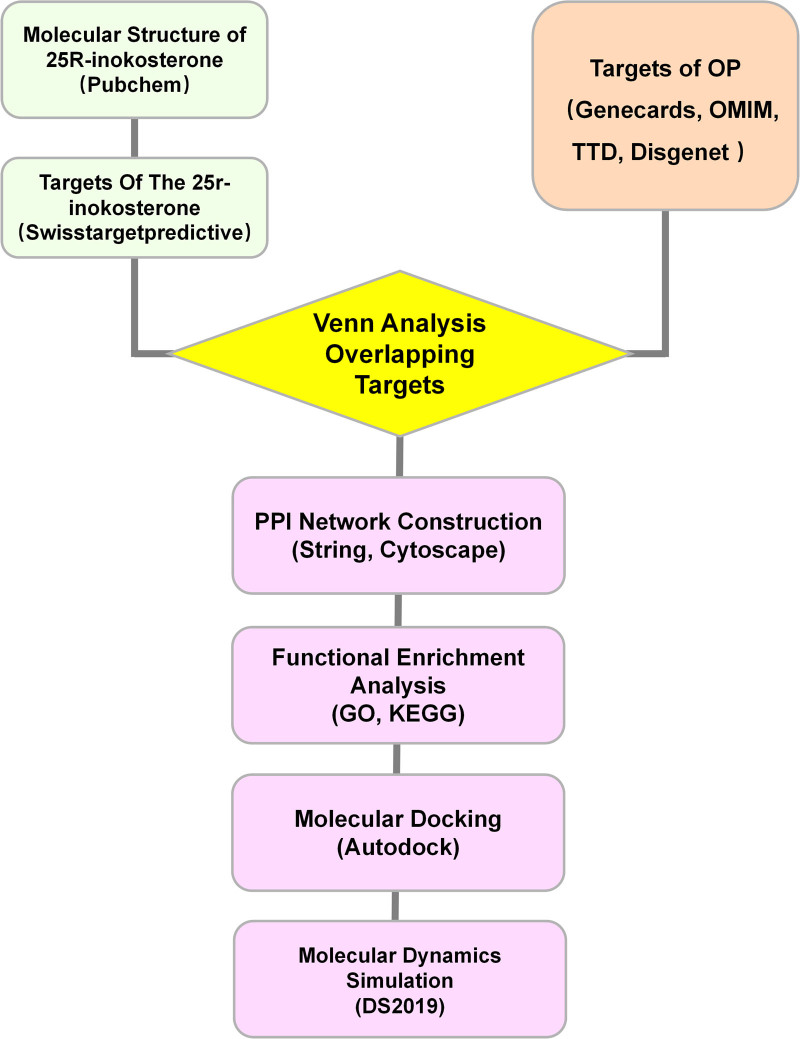
Workflow of this study.

In order to reveal the drug–disease relationship in a systematic and in-depth manner, and to provide new evidence and ideas for clinical applications and mechanism studies, this study utilized a network pharmacology^[11]^ approach to screen the potential targets of 25R-inokosterone’s anti-osteoporosis action, and explored its potential anti-osteoporosis pathway, which was combined with molecular docking^[12]^ and molecular dynamics simulations^[13]^ for preliminary validation. In Fig. 1, a workflow diagram is shown.

## 2. Methods

### 2.1. Identification of 25R-inokosterone target genes

The 2D molecular structure of 25R-inokosterone was obtained from PubChem (https://pubchem.ncbi.nlm.nih.gov/).^[14]^ The 3D structure of 25R-inokosterone saved in *mol2 format was constructed using ChemOffice software and its energy was minimized. The predicted targets of the 25R-inokosterone were obtained from SwissTargetPredictive (http://www.swisstargetprediction.ch/)^[15]^ platform, and the selection criteria were probability >0.1.

### 2.2. Screening of osteoporosis-related genes

Osteoporosis-related genes were collected using the GeneCards database (http://www.genecards.org),^[16]^ OMIM database (https://omim.org),^[17]^ therapeutic target database (https://db.idrblab.org/ttd),^[18]^ and a database of gene–disease associations (DisGeNET, http://www.disgenet.org/),^[19]^ and duplicate genes were deleted.

### 2.3. Construction of protein–protein interaction (PPI) network

The Venn diagram of the common target genes of 25R-inokosterone and OP was constructed via InteractiVenn (http://www.interactivenn.net/). The STRING (https://string-db.org/)^[20]^ was applied to construct a potential hub target gene interaction network for the common target genes by setting the protein species to “homo sapiens,” settings minimum required interaction score to “high confidence (0.700).” The PPI network model of potential hub genes was constructed by Cytoscape 3.7.2 software,^[21]^ and the cytoHubba plug-in was used to filter the hub genes, and the top 20 hub genes were ranked using the degree algorithm, the PPI networks of the top 20 hub genes based on betweenness was constructed separately.

### 2.4. Functional enrichment analysis

In order to further investigate the potential mechanism and the function of potential hub genes of 25R-inokosterone on OP, we performed gene ontology (GO) analysis by applying the clusterProfilerGO.R package and Kyoto encylopaedia of genes and genomes (KEGG) Pathway enrichment analysis by applying the clusterProfilerKEGG.R package in R language (https://www.r-project.org/) software.^[22]^ The corresponding signaling pathways were mapped with the path view package. The core pathway enrichment was analyzed according to the enrichment factor values.

### 2.5. Molecular docking analysis

The 3D structure of 25R-inokosterone was imported into AutoDockTools (v1.5.6) software^[23]^ for hydrogen atom addition and charging operations and then saved in pdbqt format. The 3D structures of the 6 central proteins associated with the signaling pathway maps and ranked high were downloaded from the PDB database (https://www.rcsb.org), the receptor proteins were dehydrated using PYMOL (v2.3.0) software, and the proteins were imported into AutoDockTools (v1.5.6) software for hydrogenation and charge calculations, and saved in the pdbqt format. Then the active pocket was searched, and the Vina script^[24]^ was run for molecular binding energy calculation and molecular docking result display. The molecular docking result output from Vina software was imported into PyMOL software for molecular docking confirmation display. If the binding energy is <0, it indicates that the ligand and receptor can spontaneously bind, and when the binding energy of Vina is ≤‐5.0 kcal mol^‐1^, it indicates that the 2 form a stable docking.

### 2.6. Molecular dynamics simulation analysis

The Simulation and Standard Dynamics Cascade modules of Discovery Studio 2019 software were used to obtain the force field parameters, and the Charm force field was used for both the ligand molecular parameters and receptor protein molecular parameters in the simulation, and the protein–ligand complexes were solvated during the calculations in the Solvation module. The protein–ligand complex was solvated during the calculation in the Solvation module. The molecular dynamics simulations were then run. The temperature of the system was increased from 50 K to 300 K, the simulation sampling time was 100 ns, and the time step was set to 0.1 ns. The process was performed on an atmospheric pressure and temperature system and the temperature was set at a constant temperature of 300 K. After the molecular dynamics calculations were completed, the structural properties of the molecular dynamics trajectories, the number of nonbonding interactions in each simulation frame, the root mean square deviations (RMSD) and root mean square fluctuations of the conformations, and nonbonding interactions formed between peptides and proteins were analyzed with the analyzer module Analyze Trajectory.

## 3. Results

### 3.1. Potential targets of 25R-inokosterone treating osteoporosis

**Figure 2.**
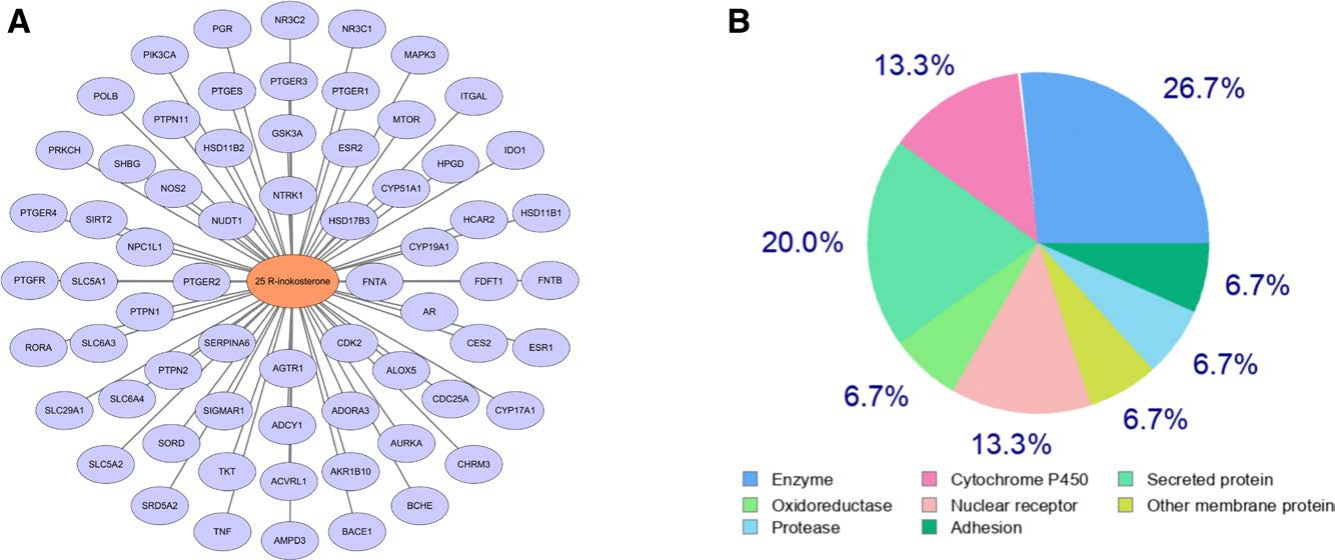
25R-inokosterone. (A) Potential target genes. (B) Target classes top 15.

The 2D and 3D structures of 25R-inokosterone were shown in Table 1. After screening, a total of 66 potential target genes of 25R-inokosterone were obtained (Fig. 2A), and target classes top 15 of 25R-inokosterone were shown in Fig. 2B.

**Table 1:**
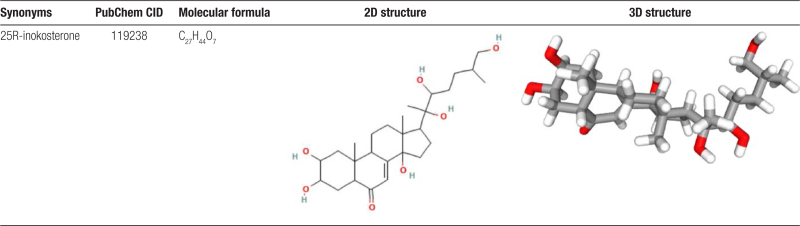
The chemical structure of 25R-inokosterone.

**Figure 3.**
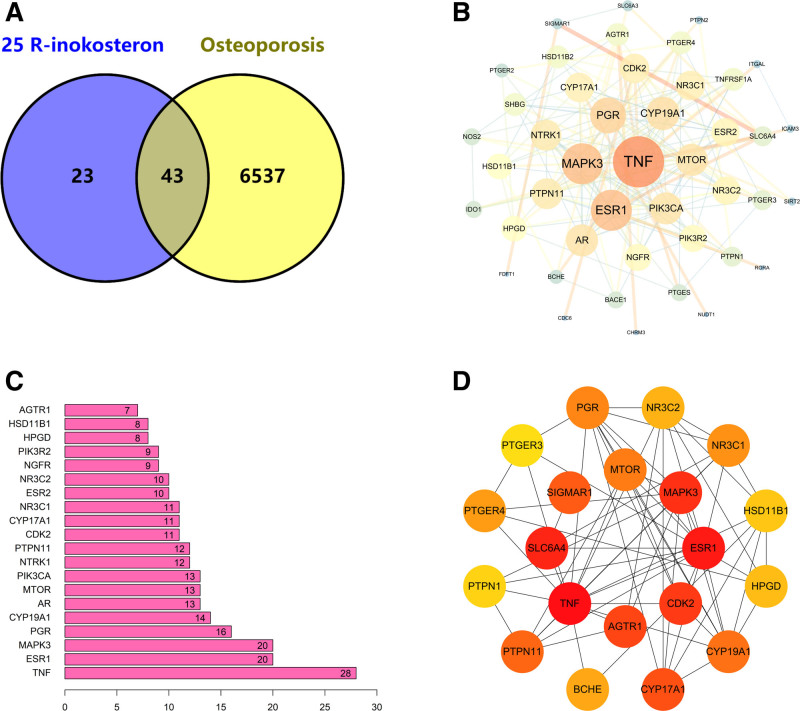
Targets of 25R-inokosterone treating osteoporosis. (A) The Venn diagram of 43 common target genes. (B) The PPI network of hub target genes. (C) Top 20 hub genes based on the degree. (D) PPI networks of the top 20 hub genes based on Betweenness. PPI = protein–protein interaction.

A total of 6580 relevant targets of OP were obtained from the GeneCards, OMIM, therapeutic target database, and DisGeNET databases. By further cross-analysis of OP and 25R-inokosterone target genes, we identified 43 potential targets of 25R-inokosterone for OP treatment (Fig. 3A).

### 3.2. PPI analysis and identification of hub genes

With STRING and Cytoscape 3.7.2 software, we constructed a PPI network of potential hub target genes of 25R-inokosterone treating osteoporosis (Fig. 3B). The top 20 hub genes were obtained by calculation based on the degree algorithm (Fig. 3C), and the PPI network of the top 20 hub genes based on betweenness was constructed separately (Fig. 3D).

### 3.3. GO enrichment analysis

**Figure 4.**
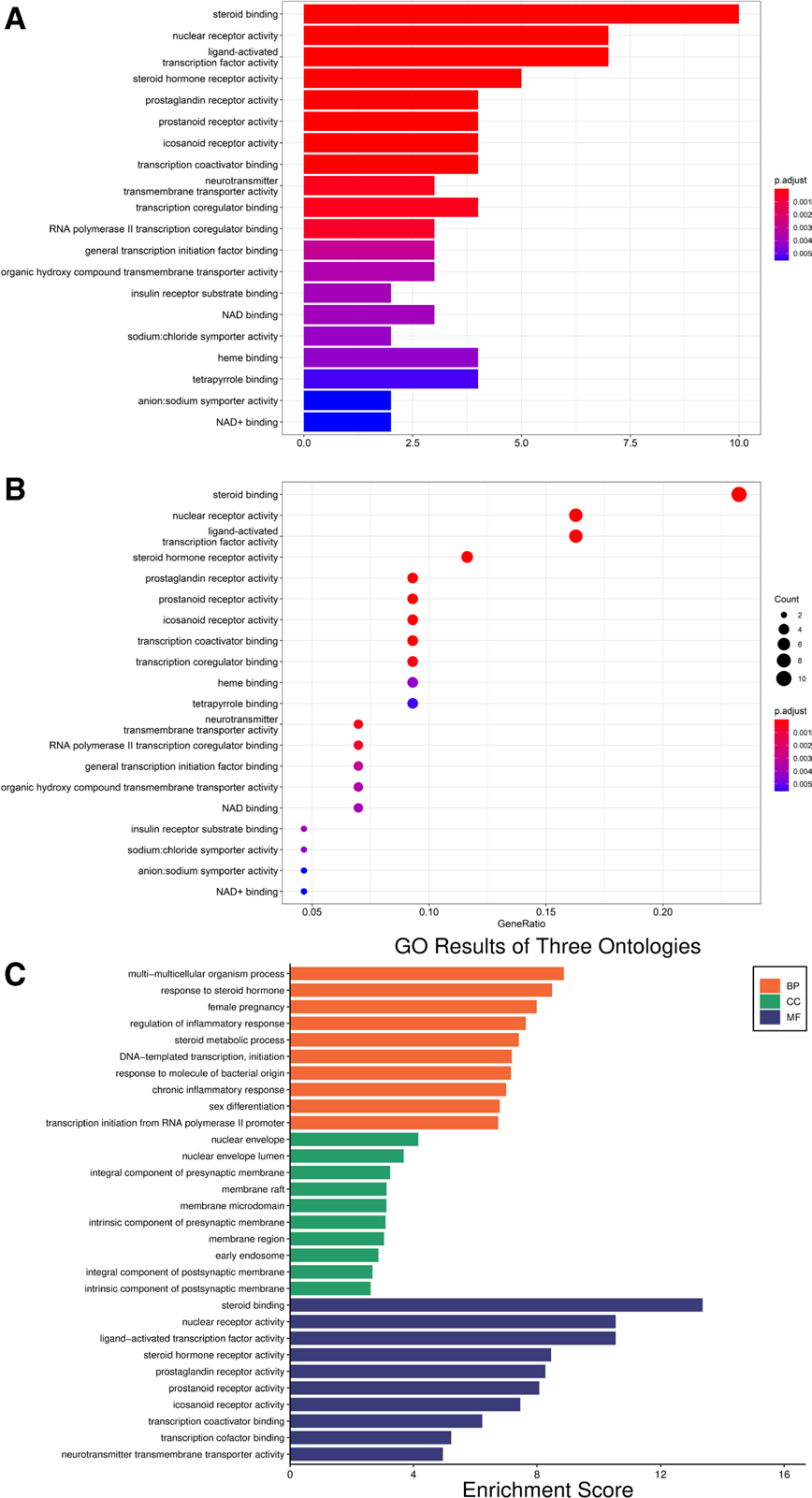
GO enrichment analyses. (A) The Bar chart of the top 20 terms. (B) The Bubble chart of the top 20 terms. (C) Top 10 significantly enriched BP, CC, and MF categories based on gene ontology. BP = biological process, CC = cellular component, GO = gene ontology, MF = molecular function.

The results showed that the GO analysis was mainly enriched in steroid binding, nuclear receptor activity, ligand-activated transcription factor activity, steroid hormone receptor activity, icosanoid receptor activity, transcription coactivator binding, etc. Biological processes were mainly focused on multi-multicellular organism processes, response to steroid hormone, and female pregnancy; cellular components were mainly concentrated in the nuclear envelope, nuclear envelope lumen, and integral component of presynaptic membrane; molecular function was mainly expressed as steroid binding, nuclear receptor activity, and ligand-activated transcription factor activity (Fig. 4A–C).

### 3.4. KEGG pathway enrichment analysis

**Figure 5.**
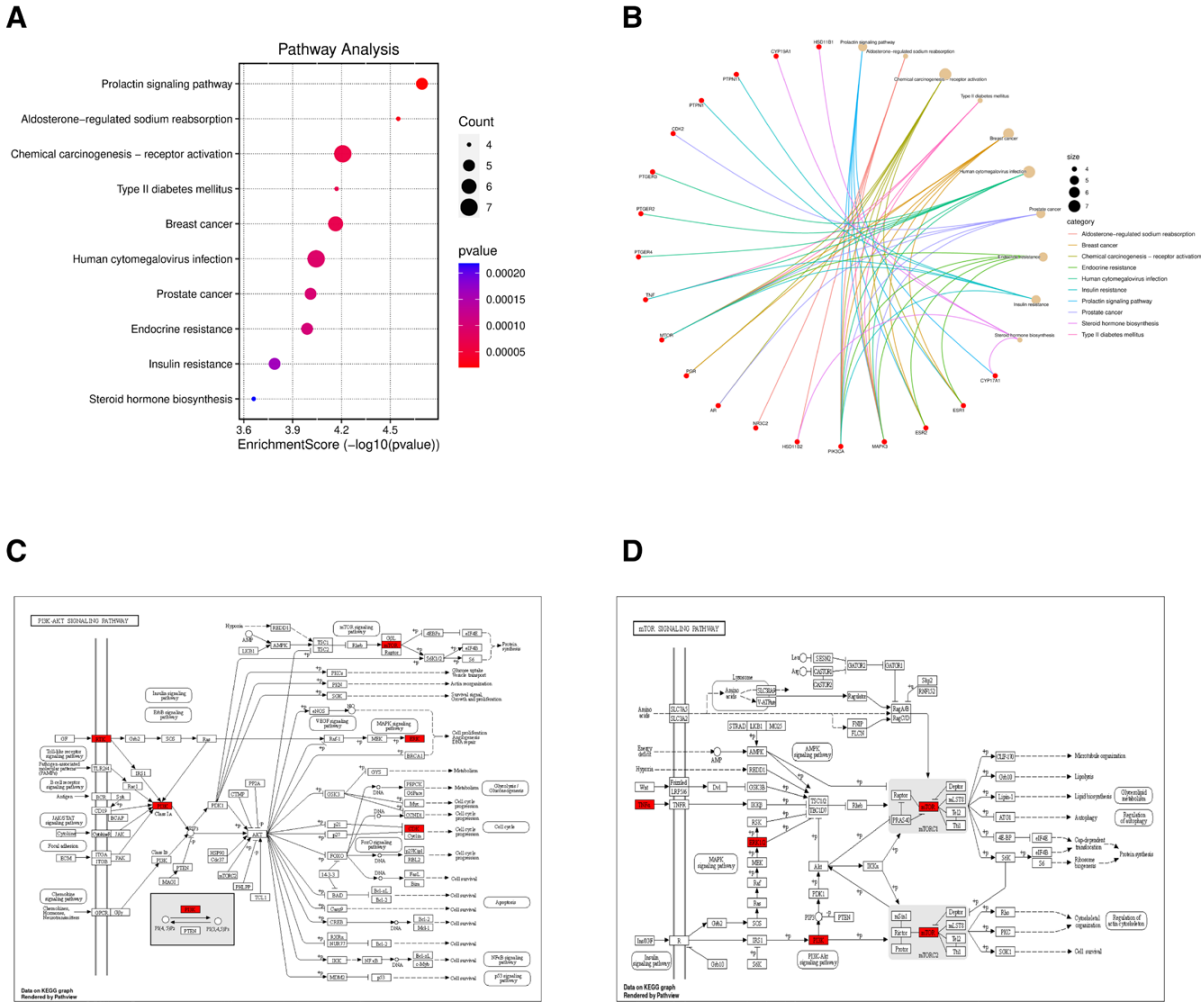
KEGG enrichment analyses. (A) The Bubble chart of the top 10 pathways. (B) Pathway cnetplot. (C) PI3K-Akt signaling pathway map. (D) mTOR signaling pathway map. KEGG = Kyoto encylopaedia of genes and genomes, PI3K = phosphatidylinositol-3 kinase.

KEGG pathway analysis revealed that it was mainly concentrated in the prolactin signaling pathway, aldosterone-regulated sodium reabsorption, chemical carcinogenesis (receptor activation), type II diabetes mellitus, breast cancer, human cytomegalovirus infection, prostate cancer, endocrine resistance, phosphatidylinositol-3 kinase (PI3K)-Akt signaling pathway, mTOR signaling pathway, and insulin resistance (Fig. 5A and B). The pathview package showed the signaling pathway diagram associated with core target genes based on count (Fig. 5C and D).

### 3.5. Molecular docking

**Figure 6.**
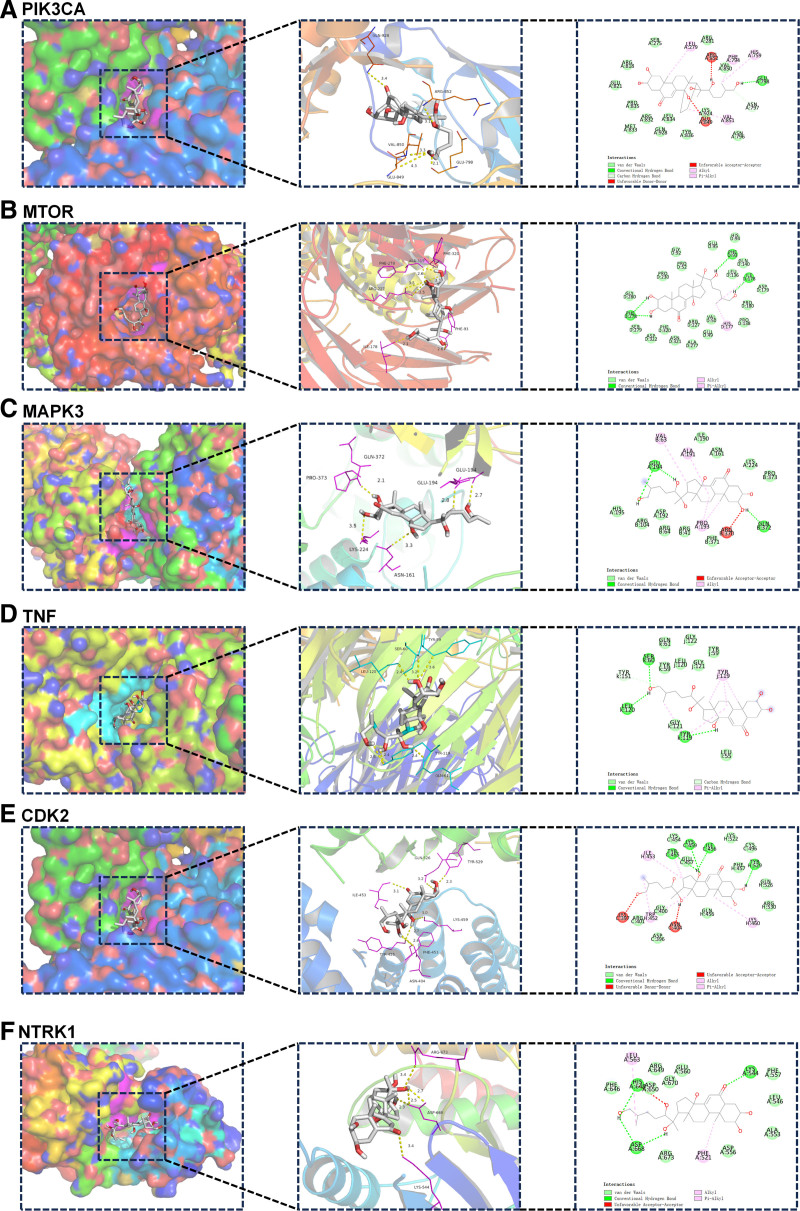
Molecular docking model of 25R-inokosterone with top 6 hub osteoporosis-related proteins.

To further validate the binding ability of 25R-inokosterone to hub targets, 25R-inokosterone was subjected to molecular docking analysis with hub proteins associated with the signaling pathway. The results of molecular docking analysis showed that 25R-inokosterone was able to bind to the active pockets of these hub proteins. Figure 6 showed the molecular docking results of the mode of action of 25R-inokosterone with 6 hub proteins. In addition, we found that the binding energies of 25R-inokosterone to the hub proteins were all <‐5.0 kcal mol^‐1^. Based on the binding energy values, 25R-inokosterone bound most strongly to PIK3CA, followed by MTOR and TNF (Table 2, Fig. 6A–F).

**Table 2:** Comparison of the binding energy of molecular docking.

Chem	Gene	PDB	Binding energy (kcal mol^−1^)
25R-inokosterone	PIK3CA	7jiu	‐8.4
	MTOR	5wbu	‐8.3
	TNF	5mu8	‐8.2
	MAPK3	4qtb	‐7.9
	CDK2	7b5l	‐7.8
	NTRK1	4pmp	‐6.9

### 3.6. Molecular dynamics simulation

For molecular dynamics simulations, the optimal conformations resulting from the docking of 25R-inokosterone with the PIK3CA/MTOR molecule were selected and solved using the explicit periodic boundary solvated water model. In addition, to analyze the physiological environment, 21,534 water, 58 sodium, and 57 chloride were added to the 25R-inokosterone–PIK3CA complex and 6771 water, 24 sodium, and 18 chloride to the 25R-inokosterone–MTOR complex.

**Figure 7.**
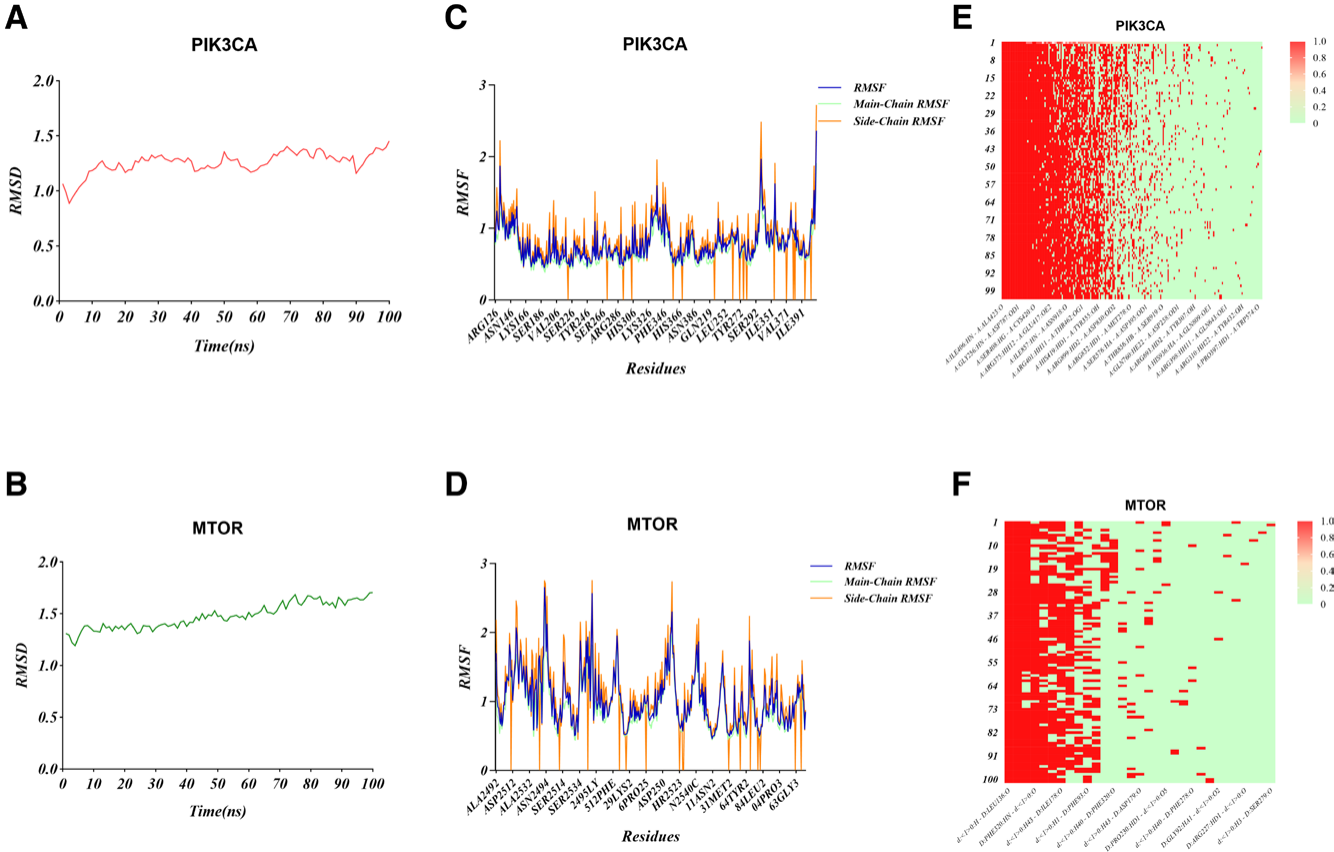
MD simulations. (A) RMSD of 25R-inokosterone–PIK3CA. (B) RMSD of 25R-inokosterone–MTOR. (C) RMSF of of 25R-inokosterone–PIK3CA. (D) RMSF of 25R-inokosterone–MTOR. (E) Hydrogen bonding heatmap of 25R-inokosterone–PIK3CA. (F) Hydrogen bonding heatmap of 25R-inokosterone–MTOR. RMSD = root mean square deviations, RMSF = root mean square fluctuations.

In order to investigate the structural stability of the protein–ligand complexes during MD simulation, the RMSD values of the complexes formed during docking were calculated for 100 ns during MD simulation, and it can be seen that the complexes of the 2 systems reached stability after 100 ns of MD simulation. In addition, the RMSD values of 25R-inokosterone–PIK3CA complex were 1.2589 ± 0.1004, and those of 25R-inokosterone–MTOR complex were 1.4782 ± 0.1216 (Fig. 7A and B). The RMSD fluctuation values of these 2 complexes were within a reasonable range, which implied that the RMSD fluctuation values of the 25R-inokosterone–PIK3CA complex were within a reasonable range and that the RMSD values of 25R-inokosterone-PIK3CA complex were within a reasonable range. inokosterone–PIK3CA and 25R-inokosterone–MTOR complexes were in a steady state throughout the MD simulation, which played a stabilizing role in the formation of the complexes. In addition, the average RMSD values of both complexes were <2.0, indicating that the binding of the 2 complexes was very stable. In order to analyze the fluctuation of various amino acids in the complexes during the MD simulation, the root mean square values of all amino acids during the simulation were calculated. The results showed that the 25R-inokosterone–PIK3CA complex fluctuated more near amino acids LYS942, LEU870, ARG524, ALA869, GLU888, LYS729, GLN871, ASP939, and LYS941, and the 25R-inokosterone–MTOR complex fluctuated more near amino acids ALA2492, TRP2549, LEU2493, ASP350, LYS2495, ASP2516, ARG310, ASP2517, ARG309, MET232, GLY2544, HIS450, and TYR2542 fluctuated more near the amino acids, while the rest of the amino acids in the complex fluctuated less, which played a role in the stability of the complex (Fig. 7C and D). The hydrogen bonding thermograms during the simulations were shown in Fig. 7E and F, from which it could be seen that the hydrogen bonding interactions were present in all conformations, indicating that these hydrogen bonds were very persistent and stable.

## 4. Discussion

Osteoporosis is the most common metabolic bone disease in the world, and it is a subclinical condition complicated by fractures, which impose significant medical and personal burdens on the patient and result in significant financial losses.^[25]^ In recent years, more and more studies have shown that many natural components in *A bidentata* Bl. can effectively exert anti-osteoporosis effects, and due to its favorable safety profile, *A bidentata* Bl. has a promising future in anti-osteoporosis.^[26–28]^ However, the mechanism of the anti-osteoporosis action of 25R-inokosterone, one of the main components of *A bidentata* Bl., has not been elucidated clearly. Therefore, we explored the target genes of 25R-inokosterone for anti-osteoporosis and analyzed their regulatory mechanism through network pharmacology, molecular docking, and molecular dynamics simulation.

In this study, we predicted and obtained the relevant targets of 25R-inokosterone and OP through several powerful databases, and after screening, we obtained 66 potential targets of 25R-inokosterone and 6580 relevant target genes of OP, and through further analysis, we obtained 43 core targets, including important hub genes, such as TNF, ESR1, MAPK3, PGR, CYP19A1, AR, MTOR, PIK3CA, NTRK1, PTPN11, and so on. Functional analysis of the hub genes showed us that GO analysis was mainly enriched in steroid binding, nuclear receptor activity, ligand-activated transcription factor activity, steroid hormone receptor activity, icosanoid receptor activity, and transcription coactivator binding. OP is a major public health problem that is primarily caused by estrogen deficiency in women after menopause, and estrogen is also a major regulator of bone metabolism in men.^[29]^ Estrogens, as one of the steroids, are a group of hormones that include estrone, estradiol (17β-estradiol), and estriol.^[30]^ Estrogens act by binding to specific estrogen receptors (ERs), including nuclear ERs (ERα and ERβ) and membrane ERs. ERα and ERβ are members of the steroid/thyroid hormone nuclear receptor superfamily, which regulate gene expression by activating transcription factors through direct binding to DNA sequences of estrogen-responsive elements that act as ligands.^[31–33]^ Estrogen regulates bone formation by modulating c-Jun N-terminal kinase activation of the SRC/SHC/extracellular signal-regulated kinase signaling pathway to inhibit osteoblast apoptosis.^[34,35]^ And estradiol inhibits osteoblast apoptosis by suppressing ITPR1 transcription and silencing information regulatory factor-2 homologue 1.^[36,37]^ Based on the results of GO enrichment analysis, we hypothesize that 25R-inokosterone may have similar effects and mechanisms of action as estrogen.

Next, in order to further investigate the possible mechanisms of 25R-inokosterone, we performed KEGG enrichment analysis on the hub gene, and the results showed that it was mainly concentrated in the PI3K-Akt signaling pathway, mTOR signaling pathway, prolactin signaling pathway, aldosterone-regulated sodium reabsorption, chemical carcinogenesis (receptor activation), and type II diabetes mellitus. The PI3K/AKT/rapamycin (mTOR) signaling pathway regulates a wide range of cellular mechanisms in mammals, including survival, proliferation, growth, metabolism, angiogenesis, and metastasis.^[38]^ It has been demonstrated that autophagy dysregulation and osteoporosis are closely linked and that autophagy activation contributes to osteogenesis and serves as a promising target for the treatment of osteoporosis.^[39]^ Existing studies have shown that the PI3K/Akt/mTOR signaling pathway is highly involved in the regulation of cellular autophagy, and the mTOR signaling pathway is a regulator that mediates the differentiation of human osteoblasts. Inhibition of mTOR phosphorylation activates autophagy while triggering antiapoptotic effects on bone marrow mesenchymal stem cells and osteoblasts. In addition, in glucocorticoid-induced osteoporosis, inhibition of the PI3K/AKT/mTOR pathway can produce anti-osteoporotic effects.^[40–42]^

Molecular docking revealed that 25R-inokosterone formed stable docking with PIK3CA, MTOR, TNF, MAPK3, CDK2, and NTRK1, which are targets involved in related signaling pathways. Among them, PIK3CA and MTOR showed the best binding activity. The mTOR complex 1 is a major integrator in a variety of pathways including autophagy, cell growth, and metabolism.^[43]^ PIK3CA activation promotes osteogenic differentiation of MAC-BMSCs through the PI3K/AKT/mTOR signaling pathway.^[44]^ The molecular docking results suggest that PIK3CA and MTOR are the core targets of 25R-inokosterone for OP treatment. This is also strongly supported by the results of molecular dynamics simulations. Taken together, it can be hypothesized that 25R-inokosterone may exert its anti-osteoporotic effects by modulating PI3K/AKT/mTOR signaling pathways.

## 5. Conclusions

In this study, through mining and screening, we identified 43 core targets of 25R-inokosterone for OP. Our findings suggest that 25R-inokosterone may against OP by regulating multiple targets such as PIK3CA, MTOR, TNF, MAPK3, CDK2, and NTRK1 and their corresponding pathways. These findings provide a theoretical basis for the treatment of OP. Moreover, we focused on the targeting of 25R-inokosterone on PIK3CA and MTOR in this paper, and other pivotal targets binding to 25R-inokosterone need to be further explored and investigated in vitro and in vivo. In the follow-up study, we plan to design pharmacological experiments to deeply probe the molecular mechanism of 25R-inokosterone for OP treatment.

## Acknowledgments

Grateful acknowledgments is made to my supervisor Professor Huo Liwei who gave me considerable help means of suggestions, comments, and criticism. In addition, I deeply appreciate the contribution to this thesis made in various ways by my coworkers.

## Author contributions

**Conceptualization:** Jun Tan, Guangwei Wang.

**Data curation:** Jun Tan, Yidong Xu, Weinian Liu, Enlong Fu, Jiling Liu.

**Formal analysis:** Jun Tan, Yidong Xu.

**Funding acquisition:** Liwei Huo.

**Investigation:** Jun Tan, Mengting Hu, Enlong Fu.

**Methodology:** Jun Tan.

**Resources:** Mengting Hu, Enlong Fu.

**Software:** Jun Tan, Mengting Hu, Enlong Fu, Jiling Liu.

**Supervision:** Jun Tan, Liwei Huo, Mengting Hu, Guangwei Wang, Jiling Liu.

**Validation:** Jun Tan, Mengting Hu, Yidong Xu, Weinian Liu, Guangwei Wang, Jiling Liu.

**Visualization:** Yidong Xu.

**Writing – original draft:** Jun Tan, Weinian Liu.

**Writing – review & editing:** Jun Tan, Liwei Huo, Weinian Liu, Guangwei Wang, Enlong Fu.
